# Editorial: Building Back Progress Towards Meeting Sustainable Development Goal 3 by 2030: Applications of AI and Digital Solutions

**DOI:** 10.3389/fpubh.2022.925483

**Published:** 2022-05-30

**Authors:** Edmond S. W. Ng, Ariel Israel

**Affiliations:** ^1^Department of Non-communicable Disease Epidemiology, Faculty of Epidemiology and Population Health, London School of Hygiene & Tropical Medicine, London, United Kingdom; ^2^Leumit Research Institute, Leumit Health Care Services, Tel Aviv, Israel

**Keywords:** artificial intelligence, machine learning, sustainable development goals, digital health, COVID−19, health service delivery

The COVID-19 pandemic has exposed the vulnerability of many health systems across the world, including some of the highest income countries, when they were overwhelmed by rapid surges of demand for health services and disruptions to the global healthcare supply chain. The suspension or even closure of some life-saving healthcare facilities and provisions, such as childhood immunization programmes, coupled with the fear of disease exposure, have made access to healthcare challenging. These factors may have led to a rise in preventable deaths due to delayed diagnosis of cancers and other diseases, and poor management of existing chronic conditions such as diabetes.

Early indications suggested that hard-won gains in global development in the last 25 years might have been lost in the first 25 weeks of the pandemic with potentially devastating impact on the progress towards the United Nations Sustainable Development Goals (SDGs) (1). In response rapid and scalable solutions became urgently needed to manage the surge in healthcare demand and mitigate the impact of the pandemic.

COVID-19 was reported to be a catalyst in one middle-income country in the African continent that helped speed up the digitalisation of healthcare and telemedicine as well as the public's adoption and acceptance of these innovations (El Otmani Dehbi et al.). The rapid adoption of digital health to mitigate the impact of COVID-19 in Morocco was facilitated by its government through legislation and amendments to existing laws. The authors showed how the deployment of digital solutions has helped address the barriers to access health services and speed up health service delivery during the pandemic.

Another paper highlighted the potential of artificial intelligence (AI) in improving eye health service delivery and thus contributing to achieving the SDGs (Sawers et al.). Drawing on examples of how commercial industry is exploiting AI to improve service delivery and an example of using machine learning (ML) algorithms to drive service delivery in eye health in Kenya by utilizing novel study designs commonly adopted in industry, the authors showed what researchers and practitioners in eye health can learn from the industry by adopting continuous testing algorithms (including their “fail fast” culture) to gain efficiency and find optimum solutions at speed to improve the delivery of known and effective eye health services and interventions. They finished with six key considerations for researchers wishing to start working with AI technology.

While AI and other digital solutions have the potential to help restore progress toward the SDGs, reliable connectivity to the internet is one key obstacle that prevents the practical deployment of such tools in the field. Mohammed et al. demonstrated how an emerging technology, known as progressive web applications (PWAs), may help obviate such hurdles. They showed how a neural network-based pneumonia mortality prediction tool developed in The Gambia could be used as a platform-independent offline PWA to assist clinical staff to triage children to hospital admission.

Monitoring and evaluation of a programme or intervention are key to our understanding of progress and success in meeting its set goals, and of any improvements that can be made. However, in the face of a global health emergency such as the COVID-19 pandemic, robust monitoring and evaluation are not always prioritized. A paper by Mason et al. showed how the rapid implementation and scale-up of eight digital tools to support public health goals in a number of low- and middle-income countries (including India, Burkina Faso, Uganda, and Vietnam) were assessed by, without any formal evaluation, adopting the mHealth Assessment and Planning for Scale Toolkit through desk research and stakeholder interviews. The authors shared three key transferable lessons from their findings that can inform the application of digital tools for other health applications.

Together the papers in this Research Topic demonstrated the critical roles that AI and other digital health tools have played during, and could potentially play beyond, the pandemic. They also showed how such tools can be assessed during rapid implementation and scale-up during a pandemic.

## Author Contributions

ESWN conceived and AI supported the topic. Both authors provided editorial inputs to the papers. All authors contributed to the article and approved the submitted version.

## Conflict of Interest

ESWN is the Senior Statistical Analyst of the Independent Advisory Committee to the Global Burden of Disease. The Committee is funded by the Bill & Melinda Gates Foundation. The remaining author declare that the research was conducted in the absence of any commercial or financial relationships that could be construed as a potential conflict of interest.

## Publisher's Note

All claims expressed in this article are solely those of the authors and do not necessarily represent those of their affiliated organizations, or those of the publisher, the editors and the reviewers. Any product that may be evaluated in this article, or claim that may be made by its manufacturer, is not guaranteed or endorsed by the publisher.
